# Auditing Antipsychotic Prescribing for First Episode Psychosis (FEP) Within Oldham Early Intervention Services

**DOI:** 10.1192/bjo.2026.11714

**Published:** 2026-06-30

**Authors:** Jade Gregson, Manish Ram, Nicola Combs

**Affiliations:** 1 Pennine Care Trust, Manchester, United Kingdom; 2 Northern Care Alliance, Manchester, United Kingdom

## Abstract

**Aims:**

Oldham Early Intervention Team (EIT) observed olanzapine was frequently prescribed as first line treatment. FEP patients may be particularly sensitive to adverse effects such as sedation and metabolic dysfunction, which can impact tolerance and adherence. NICE guidelines recommend collaborative antipsychotic prescribing with careful consideration of benefits and side effects. This audit aimed to evaluate antipsychotic prescribing patterns, assess compliance with monitoring standards, and determine whether dissemination of initial audit findings influenced prescribing practice.

**Methods:**

A retrospective clinical audit was conducted on patients accepted by Oldham EIT during two time periods: September 2023–March 2024 (initial audit, n=36) and April 2024–September 2024 (re-audit, n=33). Antipsychotic prescribing data was collected from electronic clinical records at baseline and six month follow up, alongside weekly weight monitoring over the first six weeks, in accordance with the Lester Tool. Initial audit findings were shared with Inpatient and EIT consultants prior to re-audit.

**Results:**

During initial audit, 35/36 (97%) patients were prescribed antipsychotics. Olanzapine was the most prescribed 19/35 (54%), followed by aripiprazole 12/35 (34%). Baseline weight was documented for 11/35 (31%) patients but weekly weight monitoring only for 1/35 (3%) patient.

At six-month follow-up, 31/36 (86%) patients were prescribed antipsychotics. Olanzapine remained the most prescribed 14/31 (45%), followed by Aripiprazole 9/31 (29%). Among those continuing olanzapine, 8/14 (57%) had their doses increased due to residual symptoms. 60% of those who discontinued antipsychotics had been taking olanzapine and 80% discontinued due to patient choice.

At re-audit, 31/33 (94%) patients were prescribed antipsychotics. Olanzapine remained the most prescribed, but use decreased to 11/31 (35%). Quetiapine became the second most prescribed (8/31, 26%). Baseline weight documentation improved to 20/31 (65%), though weekly weight monitoring remained low 2/31 (6%).

At six-month follow-up, 28/33 (85%) patients were prescribed antipsychotics. Aripiprazole became the most prescribed 12/28 (43%), followed by Olanzapine 7/28 (25%). One patient continuing olanzapine had their dose increased due to residual symptoms. All antipsychotic discontinuations were due to patient choice, with 80% symptom-free.

**Conclusion:**

This audit cycle demonstrated improvements in antipsychotic prescribing for FEP within Oldham EIT. Olanzapine prescribing reduced and was replaced by aripiprazole as the most prescribed antipsychotic at six month follow up. Areas for improvement were identified, with weekly weight monitoring remaining suboptimal. Furthermore, improved documentation could clarify prescribing rationale and establish if treatment refusal was linked to adverse antipsychotic effects. Continued education and re-audit are recommended to sustain improvements in prescribing practice and shared decision-making.

